# Interactions between Glycine and Glutamate through Activation of Their Transporters in Hippocampal Nerve Terminals

**DOI:** 10.3390/biomedicines11123152

**Published:** 2023-11-27

**Authors:** Katia Cortese, Maria Cristina Gagliani, Luca Raiteri

**Affiliations:** 1 Department of Experimental Medicine (DIMES), Cellular Electron Microscopy Lab, University of Genoa, 16132 Genoa, Italy; cortesek@unige.it (K.C.); gagliani@unige.it (M.C.G.); 2 Department of Pharmacy (DIFAR), Pharmacology and Toxicology Section, University of Genoa, 16148 Genoa, Italy

**Keywords:** glycine release, glutamate release, NMDA receptors, glycine transporter 1 (GlyT1), glycine transporter 2 (GlyT2), EAAT transporters

## Abstract

Evidence supports the pathophysiological relevance of crosstalk between the neurotransmitters Glycine and Glutamate and their close interactions; some reports even support the possibility of Glycine–Glutamate cotransmission in central nervous system (CNS) areas, including the hippocampus. Functional studies with isolated nerve terminals (synaptosomes) permit us to study transporter-mediated interactions between neurotransmitters that lead to the regulation of transmitter release. Our main aims here were: (i) to investigate release-regulating, transporter-mediated interactions between Glycine and Glutamate in hippocampal nerve terminals and (ii) to determine the coexistence of transporters for Glycine and Glutamate in these terminals. Purified synaptosomes, analyzed at the ultrastructural level via electron microscopy, were used as the experimental model. Mouse hippocampal synaptosomes were prelabeled with [^3^H]D-Aspartate or [^3^H]Glycine; the release of radiolabeled tracers was monitored with the superfusion technique. The main findings were that (i) exogenous Glycine stimulated [^3^H]D-Aspartate release, partly by activation of GlyT1 and in part, unusually, through GlyT2 transporters and that (ii) D-Aspartate stimulated [^3^H]glycine release by a process that was sensitive to Glutamate transporter blockers. Based on the features of the experimental model used, it is suggested that functional transporters for Glutamate and Glycine coexist in a small subset of hippocampal nerve terminals, a condition that may also be compatible with cotransmission; glycinergic and glutamatergic transporters exhibit different functions and mediate interactions between the neurotransmitters. It is hoped that increased information on Glutamate–Glycine interactions in different areas, including the hippocampus, will contribute to a better knowledge of drugs acting at “glycinergic” targets, currently under study in relation with different CNS pathologies.

## 1. Introduction

### 1.1. Transporter-Mediated Interactions between Glycine and Glutamate

Glycine (Gly) is a major inhibitory neurotransmitter (NT) in the mammalian central nervous system (CNS), and it also plays pivotal roles in excitatory neurotransmission as a coagonist of Glutamate (Glu) at the ionotropic NMDA receptor [1]. Interactions between the two amino acid NTs, Gly and Glu, are of particular pathophysiological interest and are the object of many studies (see [2,3,4,5,6,7,8,9] and references therein).

**Figure 1:**
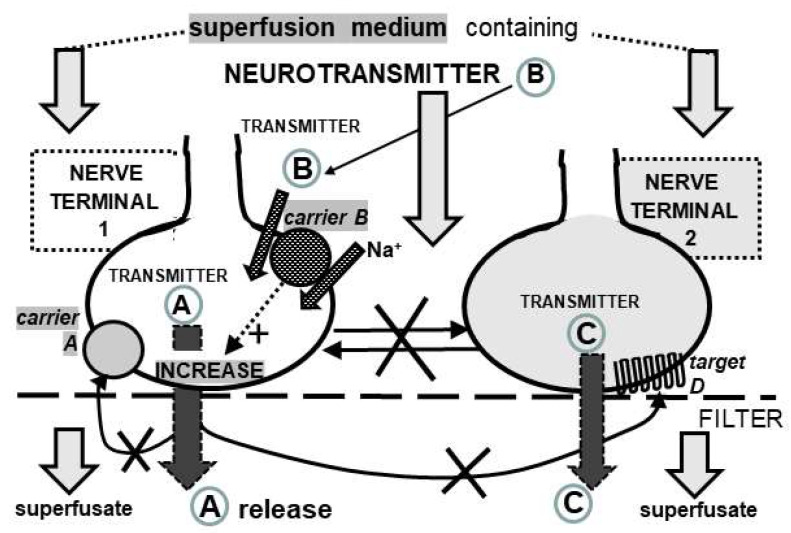
Schematic representation of the studied transporter-mediated modulation of neurotransmitter (NT) release with superfused synaptosomes. According to this technique, monolayers of synaptosomes are stratified on microporous filters and up–down superfused. In these conditions, all compounds that are released by nerve terminals (in figure, transmitter A and C, released by nerve terminal 1 and nerve terminal 2, respectively) are removed and expected to be unable to interact with targets (transporters, receptors…) located on the same or on other nerve terminals (“carrier A”, “target D”, etc.); therefore it is possible to assume that the effects of molecules released by one single nerve terminal (in figure, transmitter A and C, respectively) on other nerve terminals in the monolayer are minimized. If the release of transmitter A (measured in the superfusate) is increased by transmitter B (which, in this in vitro experimental setting, is added to the superfusion medium at appropriate concentrations), it is possible to also assume that B acts directly on the A-releasing particles (for details, see [17]). This experimental approach has permitted us to characterize the hetero-transporter-mediated modulation of NT release that is represented, in the figure, by the modulation of release of “transmitter A” by “transmitter B”. B is cotransported with Na^+^ ions in the A-releasing “nerve terminal 1” through a transporter that is selective for B (carrier B) and typically able to mediate the uptake of B; this event triggers internal pathways (dotted arrow), leading to an increase in the release of A. If such a phenomenon is detected, on the basis of the experimental technique, it is also possible to assume that functional “carrier B” proteins exist on nerve terminals that are also endowed with “carrier A”, through which radioactive transmitter A has been previously taken up ([15], review).

Interactions between two NTs include regulation of the release of one NT by another one, usually via the activation of receptors located on the releasing neurons [10,11,12,13]. Other modes of regulation of the release of a given NT (generically, “transmitter A”) can occur following activation, by a second NT (“transmitter B”), of “heterotransporters” that are selective for B, which is vehicled in the same nerve terminal which is also able to store and release A (Figure 1). The process triggers internal events, leading finally to the increase in the release of “transmitter A” (for reviews, see [14,15,16]). This mode of interaction between NTs was first established through functional studies with the technique of superfused synaptosomes [17]; subsequent immunohistochemistry studies could give support to functional data [18,19,20,21,22,23]. Figure 1 and its legend aim to present the “heterotransporter”-mediated regulation of NT release studied with the technique of superfused synaptosomes, as mentioned above, as well as some important implications, including the determination of the coexistence of functional transporters for “transmitter A” and for “transmitter B” on a same nerve terminal ([15], review).

Transporter-mediated interactions between Gly and Glu were described in nerve terminals of rodent CNSs [24]. Some important concepts could at first only be inferred, due to lack of selective pharmacological tools that were not yet available. Later on, the mechanisms of Glu release evoked by Gly through activation of Gly transporters (GlyTs) were characterized in detail in nerve terminals of mouse spinal cord [18], as were the important pathological alterations of these systems in transgenic animal models of neurodegenerative disease [22,25,26]. Of note, similar mechanisms at the nerve terminal level were observed by other groups, in particular with regard to Gly-induced Glu release in brain nerve terminals (see [27]). In spite of the often inhibitory functions exhibited by Gly (when the NT acts on strychnine-sensitive Gly receptors), the just mentioned modulation of Glu release following activation of GlyTs is always stimulatory [18,25].

### 1.2. The Possible Gly–Glu Cotransmission

As mentioned above, Gly and Glu are coagonists at NMDA receptors: however, the mechanisms through which Gly reaches NMDA receptors have remained in part elusive. A possible colocalization of Gly and Glu in nerve terminals that are able to corelease them onto NMDA receptors as cotransmitters has not been established throughout the CNS. Some reports support the view that glutamatergic and glycinergic transmission are intimately connected at the presynaptic level, particularly in the hippocampus. Müller et al. [5] found that in the hippocampus, Gly is stored in synaptic vesicles in nerve terminals that are stained for vesicular Glu transporters (vGluTs), i.e., in glutamatergic nerve terminals, and that vesicular Gly can be released during synaptic activity, possibly onto NMDA receptors. Cubelos et al. [7] purified glutamatergic (vGluT1-expressing) synaptic vesicles from the hippocampus and found that they, surprisingly, immunostained for glycine transporter 1 (GlyT1), which had been previously found by the same authors [6] in the plasma membrane of glutamatergic nerve terminals in the hippocampus. Musante et al. [28] showed that the activation of GlyT1 colocalized with NMDA autoreceptors on the plasma membrane of hippocampal glutamatergic nerve terminals exhibits a permissive role for physiological autoreceptor activation. Other research shows results that are compatible with Gly–Glu close interactions or cotransmission in different areas, including the hippocampus [29] and cerebellum [6,23]. Gly and Glu are stored together, although they are coreleased in a “segregated” way, in vesicle glutamate transporter 3 (VGluT3)-containing amacrine cells in the retina [30,31,32]. Since increasing evidence, particularly in the hippocampus, supports the hypothesis of Gly–Glu cotransmission (see also [33]), this could be further strengthened by additional functional evidence.

The coexistence on nerve terminals of transporters that are able to take up different NTs that can be costored at the intraterminal level is one of the conditions that allow us to state that the NT substrates behave as “cotransmitters” [34,35]. Functional studies have also suggested that the coexistence of transporters for two different NTs on the same nerve terminal, detected through a functional approach, can reflect, although not always, cotransmission (see [23]). Accordingly, in the spinal cord and cerebellum, where GABA and Gly coexist in the same neurons and can act as cotransmitters [36,37,38] functional transporters for GABA and Gly were found to coexist on the same nerve terminals [20,39]. Interestingly as well, and independently of cotransmission, such a functional approach highlights the interactions that can occur between transmitters (or cotransmitters).

### 1.3. Aims of the Study and Main Conclusions

In this work, the main aims were (i) to study transporter-mediated interactions between Gly and Glu (see Section 1.1) that lead to the regulation of their release in mouse hippocampal nerve terminals and add information on the transporters involved; (ii) to ascertain the coexistence of functional transporters for Gly and Glu on nerve terminals, purified from mouse hippocampi; (iii) to evaluate if the presence of the transporters under study can strengthen the idea of some kind of Gly–Glu cotransmission in this area.

It is hoped that this study can contribute to our knowledge of the functions of Gly and Glu transporters and of Gly–Glu interactions in the hippocampus.

In this regard, the main results suggest that (i) in line with previous evidence, transporters for Gly and Glu can mediate reciprocal interactions between the two NTs, leading to the modulation of their release in hippocampal nerve terminals and thus contributing to Gly–Glu crosstalk in this area; (ii) functional transporters for Gly and Glu are, indeed, present together on one (or more) subset(s) of nerve terminals, purified from mouse hippocampus; (iii) the coexistence of functional transporters for Gly and Glu can strengthen the idea of Gly–Glu cotransmission in small populations of hippocampal nerve terminals; (iv) “glycinergic” transporters of the GlyT1 type but also, surprisingly, of the glycine transporter 2 (GlyT2) type and excitatory amino acid transporters (EAATs) seem intimately connected, respectively, with glutamatergic and glycinergic neurotransmission.

## 2. Materials and Methods

### 2.1. Animals

Adult “Swiss” mice (weighing 20–25 g; Charles River, Calco, Italy) were housed in the animal facility of DIFAR, Section of Pharmacology and Toxicology, University of Genoa (authorization for animal utilization n. 484 of 8 June 2004). Animals were housed at constant temperature (22 ± 1 °C) and relative humidity (50%) under a regular light–dark schedule (lights 7.00 a.m.–7.00 p.m.). Food and water were freely available.

The animal care and experimental procedures complied with the European legislation (Directive of 22 September 2010, no. 2010/63/EU), the “ARRIVE” guidelines for reporting research, and Italian legislation and were approved by the Italian Ministry of Health (project n. 31754-1-2013). All efforts were made to minimize animal suffering and to use only the number of animals necessary to produce reliable results.

### 2.2. Preparation of Synaptosomes

Animals were sacrificed by cervical dislocation and the hippocampi were removed as quickly as possible. The tissues were homogenized in 10 volumes (100 mg/1 mL) of a solution containing 0.32 M sucrose, buffered at pH 7.4 with Tris-HCl, using a glass–Teflon tissue grinder with a clearance of 0.25 mm (12 up–down strokes in about 1 min). To obtain purified synaptosomes [40,41] the homogenate was first centrifuged (5 min, 1000× *g* at 4 °C) to remove nuclei and debris, and the supernatant was stratified on a discontinuous Percoll^®^ gradient (2, 6, 10 and 20%, *v*/*v* in Tris-buffered sucrose) and centrifuged at 33,500× *g* for 5 min. The layer between 10% and 20% Percoll^®^ (synaptosomal fraction) was collected, washed via centrifugation and resuspended in a physiological medium (standard medium), having the following composition (mM): NaCl, 140; KCl, 3; MgSO_4_, 1.2; CaCl_2_, 1.2; NaH_2_PO_4_, 1.2; NaHCO_3_, 5; glucose, 10; HEPES, 10; pH adjusted to 7.4 with NaOH (see [42]). All the above-described procedures were performed at 0–4 °C. Protein was determined according to [43], using bovine serum albumin as a standard.

### 2.3. Electron Microscopy

Synaptosomes were washed in 0.1 M cacodylate buffer and fixed in 0.1 M cacodylate buffer containing 2.5% glutaraldehyde (Electron Microscopy Science, Hatfield, PA, USA) for 1 h at room temperature. Synaptosomes were then postfixed in osmium tetroxide for 2 h and 1% uranyl acetate for 1 h. Samples were then dehydrated through a graded ethanol series and embedded in epoxy resin (Poly-Bed; Polysciences, Inc., Warrington, PA, USA) for 24 h at 60 °C. Ultrathin sections (50 nm) were cut and stained with 5% uranyl acetate in 50% ethanol. Electron micrographs were taken using a Hitachi TEM microscope (HT7800 series, Tokyo, Japan), equipped with a Megaview 3 digital camera and Radius software 2.0 (EMSIS, Muenster, Germany) [44].

### 2.4. Neurotransmitter Release Experiments

Synaptosomes were incubated at 37 °C for 15 min with [^3^H]D-aspartate ([^3^H]D-Asp; 0.03 μM) or with [^3^H]glycine ([^3^H]Gly; 0.3 µM). At the end of incubation, identical portions of the synaptosomal suspension (about 25 μg protein) were distributed as monolayers on microporous filters placed at the bottom of superfusion chambers in parallel, maintained at 37 °C (Superfusion System, Ugo Basile, Comerio, Varese, Italy), and superfused with standard medium at a flow rate of 0.5 mL/min (see [42]). After 36 min of superfusion, to equilibrate the system, four 3 min fractions were collected. Gly or D-aspartate was added at t = 39 min of superfusion. N-[(3R)-3-([1,1′-biphenyl]-4-yloxy)-3-(4-fluorophenyl)propyl]-N-methylglycine hydrochloride (NFPS), 4-benzyloxy-3,5-dimethoxy-N-[1-(dimethylaminociclopentyl)-methyl]benzamide (Org25543), strychnine, DL-threo-benzyloxyaspartic acid (DL-TBOA), and (2*S*,3*S*,4*R*)-2-Carboxy-4-isopropyl-3-pyrrolidineacetic acid (dihydrokainic acid, DHK) were added 9 min before. In one set of experiments, NFPS (0.3 µM) was present during incubation with [^3^H]Gly. In another set of experiments, Org25543 (10 µM) was present during incubation with [^3^H]Gly. Fractions collected and superfused filters were counted for radioactivity by liquid scintillation counting.

### 2.5. Data Analysis for Release Experiments

NT released in each collected fraction was expressed as a percentage of the radioactivity content of synaptosomes at the start of the respective collection period (fractional rate × 100). Drug effects were evaluated by calculating the ratio between the efflux in the third fraction collected and that of the first fraction. This ratio was compared with the corresponding value calculated under control conditions.

Statistical analysis of data was performed, when appropriate, through one-way ANOVA followed by Dunnett’s test. Differences between means were considered statistically significant for *p* < 0.05.

### 2.6. Chemicals

[^3^H]D-Aspartate (specific activity: 38 Ci/mmol) and [^3^H]glycine (specific activity: 20 Ci/mmol) were purchased from Perkin Elmer (Boston, MA, USA). Percoll^®^, glycine, D-aspartate, and strychnine were from Sigma Chemical Co. (St. Louis, MO, USA). DHK, DL-TBOA, and NFPS were from Tocris Bioscience (Bristol, UK). Org25543 was originally a gift from Dr. Hardy Sundaram (Organon Laboratories Ltd., Newhouse, UK).

## 3. Results

### 3.1. The Experimental Model: Ultrastructural Analysis

**Figure 2:**
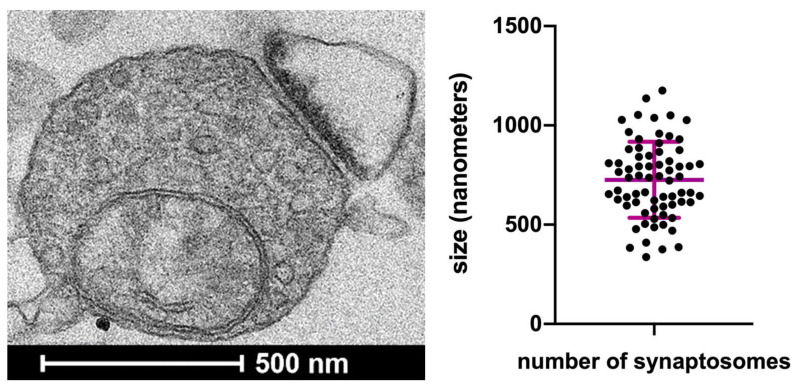
(**Left panel**). Ultrastructural analysis of purified synaptosomes via electron microscopy (EM). The micrograph depicts a single synaptosomal particle (see text). The higher-density part (top right) represents the postsynaptic density. Scale bar: 500 nm. The figure is representative of the EM analysis of *n* = 5 different synaptosomal preparations. (**Right panel**). The analysis of synaptosome size was conducted based on a comprehensive examination of *n* = 10 electron micrographs and plotted as scatter dot plot. A total of *n* = 72 individual synaptosomes were measured, revealing a nuanced distribution of sizes ranging from 337 nm to 1175 nm. The mean synaptosome size was determined to be 706 nm (violet horizontal line), with a median size of 730 nm. The standard deviation (SD) in synaptosome sizes was calculated as 224 nm (vertical line), indicating the degree of variability within the measured population.

Isolated nerve terminals (synaptosomes) were chosen as the experimental model for the present study. The preparations of purified synaptosomes were analyzed at the ultrastructural level via electron microscopy (EM). In Figure 2, the left panel is a representative image that shows a round-shaped structure limited by a membrane, which exhibits a diameter around 500–1000 nM, multiple synaptic vesicles, and a mitochondrion that is consistent with the structure of a single synaptosome (see also [45,46,47]). The upper right, higher-density part in Figure 2 represents the “postsynaptic density” that is often associated with synaptosomal particles but has no relevance in studies of NT release performed by exploiting the “superfusion technique” that is also used in this study, as outlined below (see [48], p. 3, and references therein).

### 3.2. Effects of Gly and of Selective GlyT Blockers in Hippocampal Glutamate-Releasing Nerve Terminals

**Figure 3:**
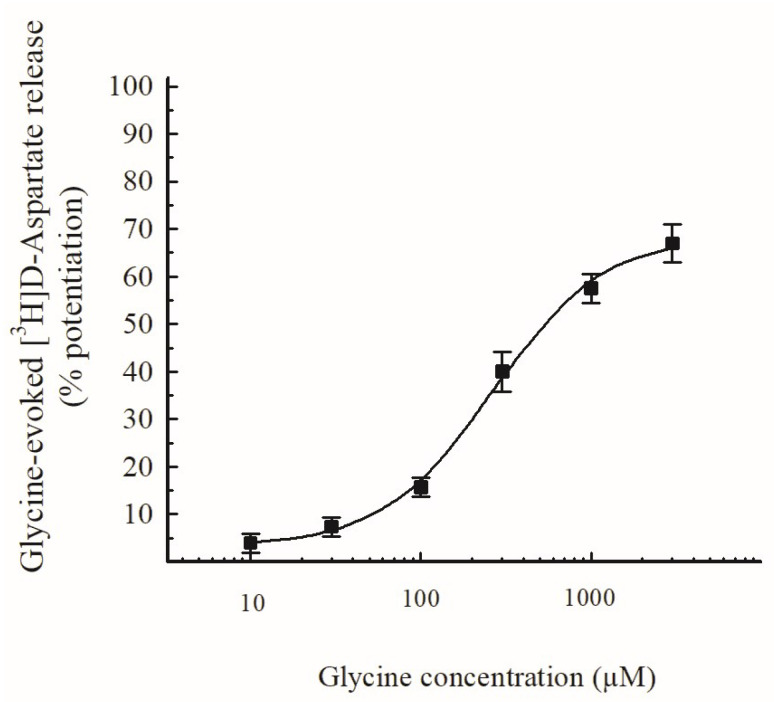
Release of [^3^H]D-Aspartate ([^3^H]D-Asp) evoked by different concentrations of Glycine (Gly) from purified mouse hippocampal synaptosomes. Synaptosomes were exposed in superfusion to varying concentrations of Gly. The effect of Gly was evaluated by performing the ratio between the efflux in the third fraction collected and that in the first fraction. This ratio was compared with the corresponding ratio obtained under control conditions. Results are expressed as percentage potentiation with respect to the basal efflux. The fraction of intraterminal [^3^H]D-Asp released under control conditions amounted to 0.23 ± 0.02% of the total tritium content per minute. Data represent means ± SEM of 3–5 experiments performed in triplicate (three superfusion chambers for each experimental condition).

To characterize the Gly-induced regulation of Glu release, synaptosomes that were purified from mouse hippocampi were prelabeled with [^3^H]D-Aspartate ([^3^H]D-Asp; see Section 2.4) and exposed in superfusion to Gly. Figure 3 shows that the exogenously added Gly increased the spontaneous [^3^H]D-Asp release in a concentration-dependent manner, exhibiting an EC_50_ value around 250 µM.

**Figure 4:**
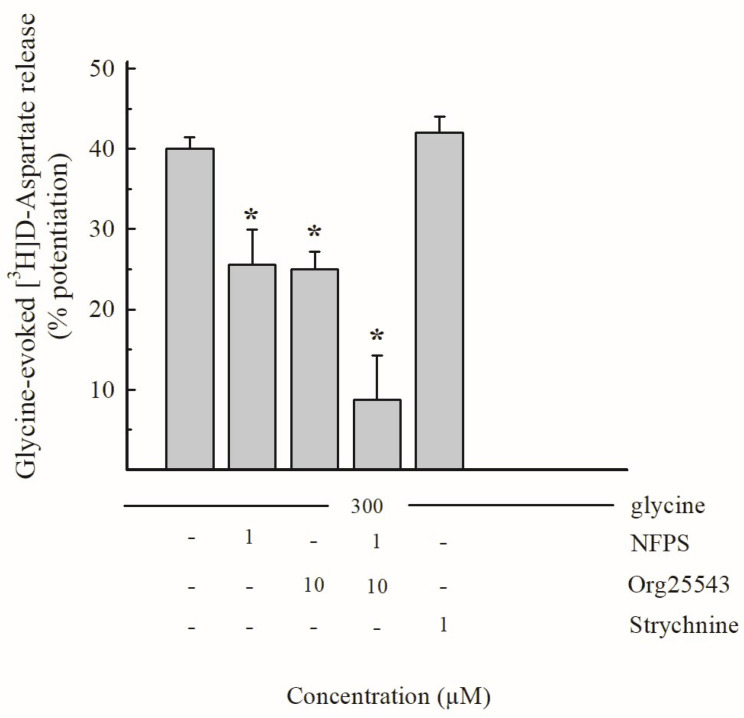
Effects of the selective Glycine transporters (GlyTs) blockers NFPS and Org25543 and of the Gly receptor antagonist strychnine on the release of [^3^H]D-Asp evoked by Gly from mouse hippocampal synaptosomes. Drugs were introduced 9 min before Gly. Results are expressed as percentage potentiation with respect to the basal efflux. Means ± SEM of 3–4 experiments in triplicate are reported. * *p* < 0.05 versus the control value which represents the effect of Gly in the absence of drugs (one-way ANOVA followed by Dunnett’s test).

The effect of 300 µM Gly (percent potentiation over the basal release: 40.0 ± 4.2%; Figure 3) was tested in the presence of drugs acting at glycinergic receptors or transporters: as shown in Figure 4, the effect was partially sensitive to the selective GlyT1 transporter blocker NFPS (1 µM; percent inhibition 35%) and partially to the selective GlyT2 transporter blocker Org25543 (10 µM; percent inhibition around 35% as well). When added together, the two inhibitors NFPS and Org25543 strongly inhibited the effect of Gly in a manner that exhibits additivity (total percent inhibition of the effect of Gly about 80%; Figure 4). Finally, the effect of 300 µM Gly was insensitive to the ionotropic glycine receptor antagonist strychnine (1 µM; Figure 4). Considering the characteristics of the superfusion technique (see [15] and Section 4.1), the results reported in Figure 3 and Figure 4 suggest that Gly enhances the release of [^3^H]D-Asp largely following direct interaction with, and activation of, transporters of the GlyT1 and GlyT2 type, which are located on nerve terminals that are able to release [^3^H]D-Asp, which possesses glutamatergic EAATs, since they have previously taken up the radioactive tracer.

### 3.3. Effects of D-Asp and of EAAT Blockers on the Release of [^3^H]glycine

**Figure 5:**
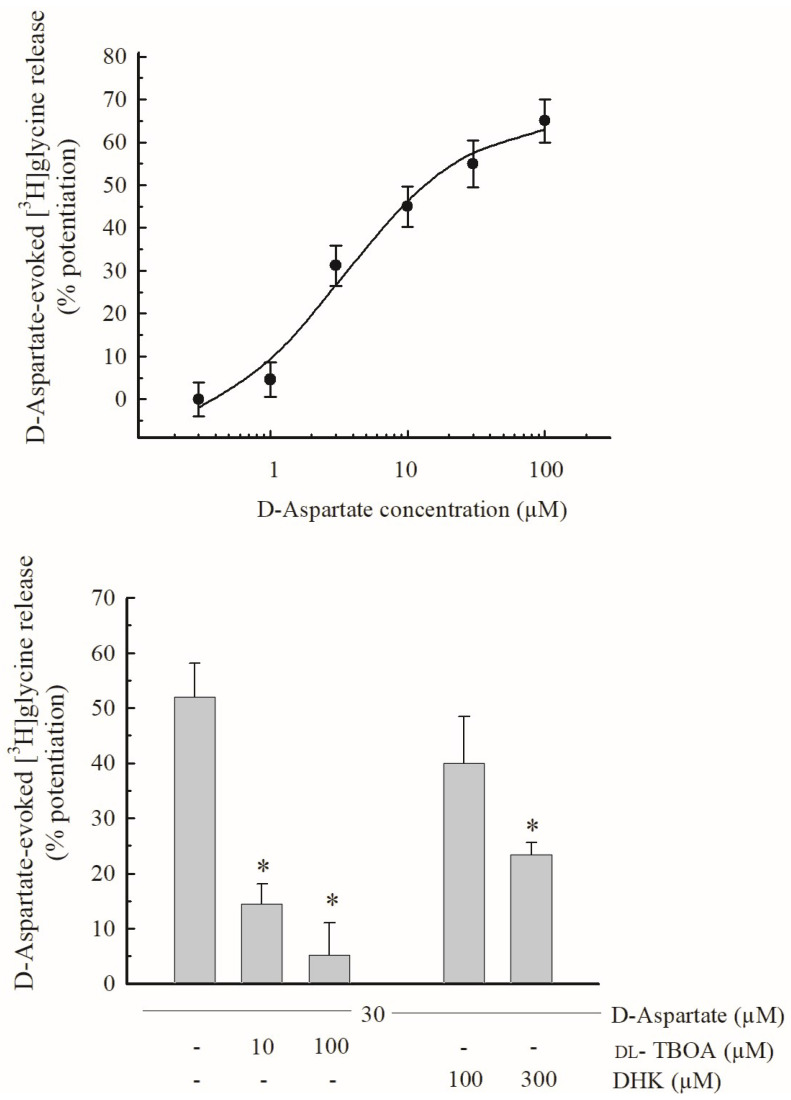
(**Upper panel**): Release of [^3^H]Glycine ([^3^H]Gly) evoked by different concentrations of D-Aspartate (D-Asp) from purified mouse hippocampal synaptosomes. Synaptosomes were exposed in superfusion to different concentrations of D-Asp. The effect of D-Asp was evaluated by performing the ratio between the efflux in the third fraction collected and that in the first fraction. This ratio was compared with the corresponding ratio obtained under control conditions. Results are expressed as percentage potentiation with respect to the basal efflux. Data are means ± SEM of 3–5 experiments in triplicate. (**Lower panel**): Effects of the Excitatory Amino Acid Transporters (EAATs) blocker DL-TBOA and of the selective EAAT_2_ transporter blocker dihydrokainate (DHK) on the release of [^3^H]Gly evoked by 30 µM D-Asp from mouse hippocampal synaptosomes. D-Asp was added at t = 39 min of superfusion. Drugs were introduced 9 min before D-Asp. Results are expressed as percentage potentiation with respect to the basal efflux (means ± SEM). * *p* < 0.01 versus the control value which represents the effect of D-Asp alone (one-way ANOVA followed by Dunnett’s test).

In another set of experiments, mouse hippocampal synaptosomes were prelabeled with [^3^H]Gly (0.3 µM) and exposed in superfusion to D-Asp (see Section 2.4). D-Asp was used here as a substrate of glutamatergic EAATs. Figure 5 (top panel) shows that D-Asp increased the release of [^3^H]Gly in a concentration-dependent manner, exhibiting EC_50_ = 5 µM and E_max_ about 65%. Figure 5 (bottom panel) also shows that the effect of D-Asp (30 µM) was strongly inhibited by 10 µM DL-TBOA, a blocker of EAATs, and almost completely prevented by 100 µM of the compound. Finally, Figure 5, bottom panel also shows that the effect of D-Asp on the release of [^3^H]Gly was slightly, although not significantly, inhibited by 100 µM dihydrokainate (DHK), a selective EAAT_2_ transporter blocker [49], and halved by 300 µM of the compound.

Similarly to the Gly-evoked release of [^3^H]D-Asp (Section 3.2), these observations suggest that D-Asp triggers [^3^H]Gly release directly through the activation of EAATs, at least as initial targets (but see Section 4.4), and that these EAATs are located on hippocampal nerve endings that also express GlyTs.

### 3.4. Selective Labeling through GlyT1 or GlyT2 and Effects of D-Asp on [^3^H]glycine Release

**Figure 6:**
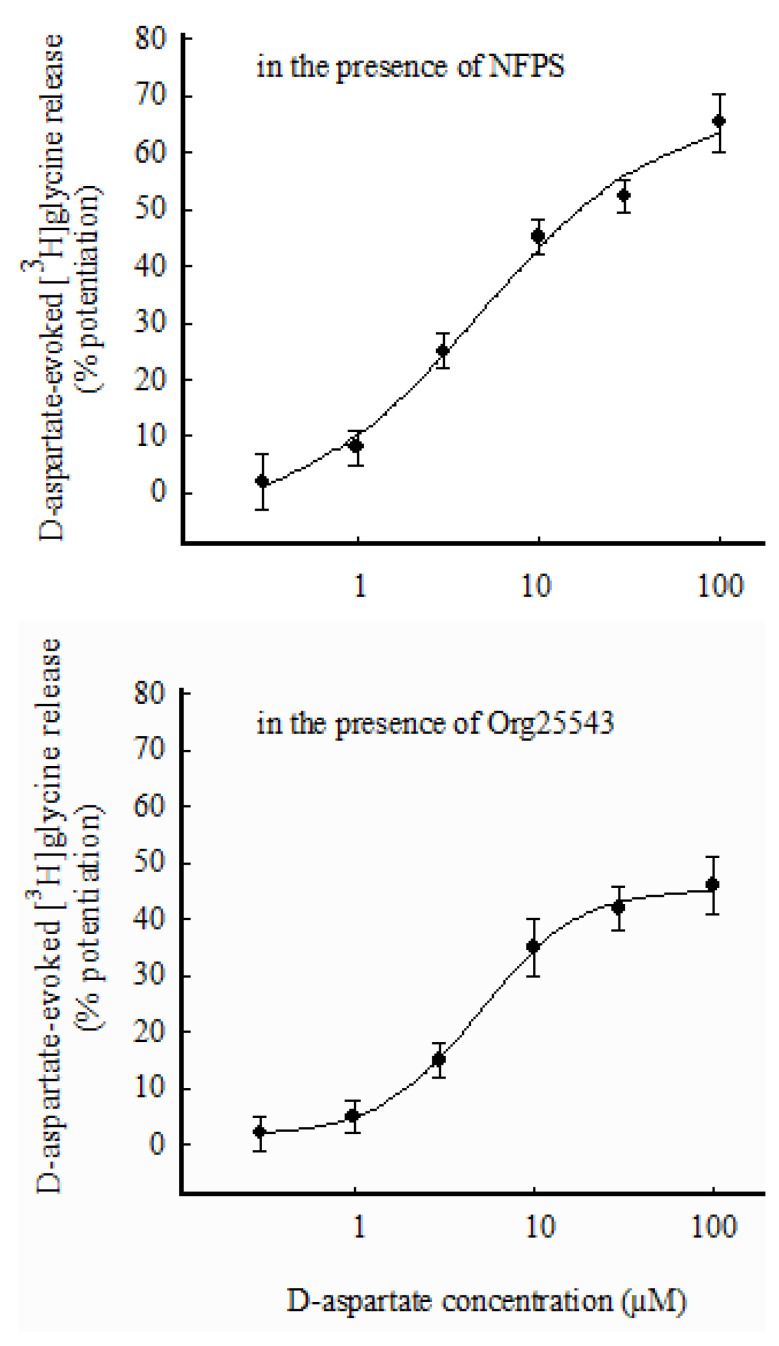
(**Top panel**): Release of [^3^H]Gly evoked by different concentrations of D-Asp from purified mouse hippocampal synaptosomes exposed to NFPS (0.3 µM) during prelabeling with the radioactive tracer. Synaptosomes were exposed in superfusion to different concentrations of D-Asp. The effect of D-Asp was evaluated by performing the ratio between the efflux in the third fraction collected and that in the first fraction. This ratio was compared with the corresponding ratio obtained under control conditions. Results are expressed as percentage potentiation with respect to the basal efflux. The fraction of intraterminal [^3^H]Gly released under control conditions amounted to 2.07 ± 0.25% of the total tritium content per minute. Data are means ± SEM of 3–5 experiments in triplicate. (**Bottom panel**): Release of [^3^H]Gly evoked by different concentrations of D-Asp from purified mouse hippocampal synaptosomes exposed to Org25543 (10 µM) during prelabeling with the radioactive tracer. Synaptosomes were then exposed in superfusion to different concentrations of D-Asp. The effect of D-Asp was evaluated by performing the ratio between the efflux in the third fraction collected and that in the first fraction. This ratio was compared with the corresponding ratio obtained under control conditions. Results are expressed as percentage potentiation with respect to the basal efflux. The fraction of intraterminal [^3^H]Gly released under control conditions amounted to 1.97 ± 0.24% of the total tritium content per minute. Data are means ± SEM of 3–5 experiments in triplicate.

A further set of experiments was performed, where synaptosomes were prelabeled with [^3^H]Gly (0.3 µM) in the presence of the selective GlyT1 transporter blocker NFPS (0.3 µM) to label synaptosomes preferentially through GlyT2. As shown in Figure 6 (top panel), D-Asp increased the release of [^3^H]Gly in a concentration-dependent manner, exhibiting EC_50_ about 5 µM and E_max_ = 70%. Finally, in a last group of experiments, mouse hippocampal synaptosomes were prelabeled with [^3^H]Gly (0.3 µM) in the presence of the selective GlyT2 transporter blocker Org25543 (10 µM) to obtain selective labeling through GLYT1. Figure 6 (bottom panel) shows the potentiation by D-Asp of the release of [^3^H]Gly from this preparation. The effect was concentration-dependent, with EC_50_ about 5 µM and E_max_ = 45%.

## 4. Discussion

### 4.1. Considerations on the Experimental Model and Technique

Synaptosomes are “pinched-off” nerve terminals which represent a widely recognized in vitro experimental model to study presynaptic events, including the mechanisms and regulation of NT release ([12,48,50]and references therein). This model was therefore chosen to study presynaptic Gly–Glu interactions and the coexistence of functional transporters for Gly and Glu.

To investigate the effects of Gly on Glu release, synaptosomes were labeled with [^3^H]D-Asp, a relatively non-metabolizable analogue of Glu that is frequently used in studies of NT release as a good marker of endogenous Glu (see [51,52]). The release of radiolabeled transmitters (Section 3.2 and Section 3.3) was monitored with the superfusion technique, an experimental approach that has long been recognized as highly appropriate for studying the presynaptic mechanisms of NT release [15,53,54,55,56,57,58]. Advantages of this approach include the minimization of indirect effects and the possibility to highlight direct effects of the added substances on radio-labeled synaptosomes, thus allowing us to support the interpretations of results shown in Figure 3, Figure 4 and Figure 5 (see Section 3.2 and Section 3.3).

It is known that preparations enriched in synaptosomes, although purified, can still contain residual particles of glial origin, which are able to take up and subsequently release different NTs. Confocal microscopy experiments with our preparations of synaptosomes purified from mouse spinal cords and hippocampi [42,59] permitted us to establish that no more than 10, max 15%, of the particles present in “purified” synaptosomal preparations can still be identified as “gliosomes” of astrocytic origin [60]. However, according to [49], in the same preparation enriched in synaptosomes, most of the [^3^H]D-Asp uptake should occur preferentially in nerve terminals (see [49]). For similar reasons, monitoring [^3^H]Gly release could permit us to preferentially measure the release of the amino acid from particles (that have previously accumulated it through GlyTs) of neuronal origin. Nevertheless, some caution should be practiced, since a certain degree of contribution of glial particles that are able to take up the two tritiated amino acid transmitters and, later, release them during superfusion cannot be completely excluded.

### 4.2. Localization and Functions of GlyTs on Hippocampal Glutamate-Releasing Nerve Terminals

The results outlined in Section 3.2 suggest that Gly enhances [^3^H]D-Asp release following activation of GlyT1 and GlyT2 transporters, located on hippocampal nerve terminals that also express EAATs. The calculated EC_50_ value (about 250 µM; Figure 3) is comparable (in the same, or a close order of magnitude) with Km values for Gly uptake reported in different studies [61,62,63,64]. This value can seem far from physiological; however, it is reported that similar values also have pathophysiological relevance [65,66]. Moreover, it exhibits similarity with previously calculated EC_50_ values for hetero-transporter-mediated effects of Gly (Gly-evoked GABA release from nerve terminals of spinal cord [20] and cerebellum [21]).

As shown in Figure 4, the concomitant addition of the selective GlyT1 and GlyT2 blockers counteracted the effect of Gly by ~80%, and strychnine was ineffective. The residual, minor effect of Gly, which is insensitive to blockers of GlyTs, might reflect that targets other than GlyTs are also involved. Of note, Gly is a coagonist of Glu at NMDA receptors. However, the superfusion medium used is free of significant amounts of Glu or other NMDA agonists; these same compounds, likely released by synaptosomal particles, are quickly removed through superfusion [15], which should avoid or minimize their interaction with targets (including NMDA receptors) located on the “superfused” synaptosomes. Accordingly, we propose that NMDA receptors are not involved here as initial targets of the effect of Gly. An involvement of other transport systems that are reported to vehicle Gly with a high affinity (see [9,67]) might be possible, although this further point is not within the aims of the study.

**Figure 7:**
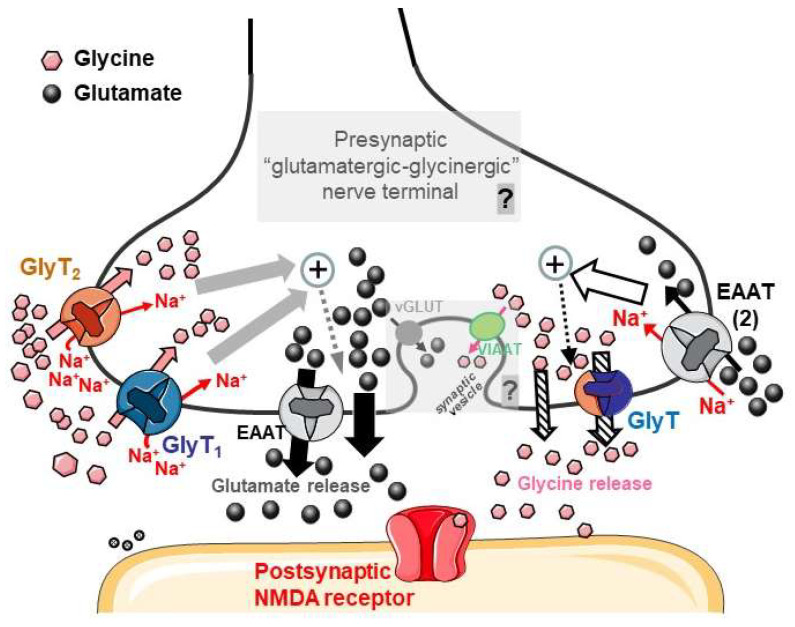
Representation of the transporter-mediated interactions between Glycine and Glutamate in mouse hippocampal nerve terminals based on the conclusions of this work and other available research. The present figure is placed in the context of the hypothesis of Gly–Glu cotransmission, which we believe is strengthened by our observations. Independently of cotransmission, the two interactions described as A and B (see below) are here illustrated in the same figure, although their coexistence in the same nerve terminal is still speculative.

As to the subset of nerve terminals on which GlyTs coexist with EAATs, it is suggested that these particles represent, at least in part, the glutamatergic nerve terminals endowed with GlyT1 transporters that are identified in the hippocampus by Cubelos et al. [6,7]. To summarize, the GlyTs studied here exhibit at least two functions: (i) to take up Gly and remove its excess from synaptic space and (ii) to enhance the release of Glu, as part of the fine functional interaction between Gly and Glu. In addition, (iii) a third function of these GlyTs could be speculated, considering also the results of other research [5,6,7]: due to their accumulative power, these GlyTs could lead to Gly possibly being costored with Glu as a cotransmitter in a proposed “mixed glutamatergic-glycinergic” terminal (see also Section 4.3 and Section 5 and Figure 7).

### 4.3. Considerations on the GlyT Types Found on Mouse Hippocampal Glu-Releasing Nerve Terminals

In the CNS, the two most relevant high-affinity transporters for Gly are known, namely, GlyT1 and GlyT2. GlyT1, originally considered almost exclusively as “glial” transporters, are also present in neurons, particularly in glutamatergic neurons, in areas including the hippocampus [6,7,9,28,29,68,69]. If the presence of GlyT1 in glutamatergic neurons has been well demonstrated, the functional data suggesting that GlyT2 transporters exist in some Glu-releasing nerve terminals in the hippocampus seem very unusual and deserve some comments. First, to the best of our knowledge, GlyT2 expression in the hippocampus is still a matter of debate (see [9]). Second, it is widely accepted that GlyT2 are almost exclusively located on classical glycinergic nerve terminals; accordingly, they are considered a good marker of glycinergic boutons [70,71,72,73,74,75] and their presence on glutamate-releasing terminals can seem unlikely.

With regard to the regional expression of GlyT2, different morphological studies have, indeed, reported that GlyT2 was restricted to the spinal cord, brainstem and cerebellum, being essentially undetectable in the hippocampus [70,76,77]. However, some reports show (or are compatible with) the presence of GlyT2 in this area as well. Interneurons in the hippocampus were found to be immunopositive, although weakly, for GlyT2 [78,79]. Nerve terminals from telencephalic regions were found to be able to perform uptake of Gly in a way that is sensitive to GlyT2 inhibitors, although the GlyT2-mediated uptake was found to be the lowest in the hippocampus [80]; very similar functional results, together with confocal microscopy evidence of the presence of GlyT2, were observed by Aroeira et al. [81] in rat hippocampal synaptosomes.

With regard to the “exceptional” presence of GlyT2 in CNS cells other than “classical” glycinergic neurons, this is reported or hypothesized by only very few studies. Recent reports could find GlyT2 transporters in glial cells [82,83]; a possible GLYT2 labeling in GABAergic neurons has been indicated [84]; and functional and immunohistochemical evidence permitted researchers to detect GLYT2 in mouse spinal cord Glu-releasing nerve terminals (immunostained for vGLUT1) [18,22] and in GABAergic nerve terminals [20]. Some further reports indicate a possible sparse GlyT2 presence in excitatory neurons (see [33], p. 99 and references therein). Besides this, to the best of our knowledge, morphological, immunochemical and other studies tend to exclude the presence of GlyT2 in “glutamatergic” nerve terminals, in particular in the hippocampus. The possible, at least partial, explanations of this discrepancy might include, in our opinion, different considerations:

(i) It was proposed that even a few GlyT2 transporters, barely detectable through morphological approaches, can accumulate enough Gly that it can be measured by functional uptake assays [80], possibly also due to the very efficient accumulative power associated with GlyT2-mediated uptake: as established by Roux and Supplisson [85], GlyT1 has a stoichiometry of 2Na^+^/Cl^−^/Gly, while the stoichiometry of GlyT2 was reported to be 3 Na^+^/Cl^−^/Gly, so that the driving force for Gly uphill transport is much larger with GlyT2 than GlyT1 [86]. Similarly, we suggest that a few GlyT2 on a possibly small subset of hippocampal nerve terminals, able to release [^3^H]D-Asp/Glu, are activated by Gly due to their strong accumulative power with enough efficiency to elicit a measurable functional response in experiments in which the release of preloaded [^3^H]D-Asp induced by Gly is monitored (present work).
(ii) According to the considerations above, the synaptosomal subpopulation considered here could represent a very low percentage of the entire population of nerve terminals; in these conditions, both an advantage and a caveat of this preparation become evident: advantages of synaptosomes include the possibility of obtaining functional results even when the targets under study are poorly expressed in a certain CNS area [23,58]. While it is expected that functional results should be confirmed by immunochemical data that show the presence of the target structures under study, unfortunately, due to the reasons just discussed, this might be hard or impossible if such targets (for example, GlyT2 on Glu-releasing terminals) are poorly expressed (see [58], p. 1003).

To conclude, our data suggesting the existence of GlyT2 on Glu-releasing (“glutamatergic”) nerve terminals are unusual and should to date be considered with caution although some explanations can be given. As speculated above (Section 4.2; see also Figure 7 and legend), a “proposed” subset of nerve terminals in which Glu and Gly are present together might perhaps exhibit “mixed” glutamatergic–glycinergic features that can include the typically “glycinergic” GlyT2.

### 4.4. Localization and Functions of EAATs on Hippocampal Nerve Terminals That Release [^3^H]glycine and Considerations of Possible Involvement of Other Glutamatergic Targets

In the experiments of [^3^H]Gly release (Section 3.3 and Section 3.4), D-Asp, a known substrate of EAATs, was used instead of Glu to preferentially mimic Glu in transporter activation. The results suggest that EAATs located on hippocampal nerve terminals that are also endowed with GlyTs can trigger [^3^H]Gly release if activated by substrates like D-Asp (and endogenous Glu); it is also suggested that the EAATs involved include EAAT_2_-type transporters, because the effect of D-Asp was in part sensitive to DHK, a selective EAAT_2_ blocker (Section 3.3). The calculated EC_50_ values (Figure 5, top panel; but see also Figure 6) for D-Asp in this system (5 µM) are not far from the Km values for high-affinity Glu uptake in CNS tissues reported in early studies [87,88]; moreover, they exhibit a similarity with EC_50_ values reported for hetero-transporter-mediated effects of Glu and of D-Asp previously observed (D-Asp-evoked release of GABA; [89]).

It must be emphasized, however, that besides its activity as an EAATs substrate, D-Asp is also an effective agonist at NMDA receptors, and that presynaptic NMDA can trigger NT release. The activation of NMDA by agonists requires the concomitant presence of the coagonist glycine or D-serine; actually, physiological solutions contain some unavoidable glycine contamination (at least 40–60 nM; [90]) that could permit receptor activation. Usually a depolarizing stimulus, able to remove Mg^2+^ ions that block the NMDA receptor-associated channel, should also be required to activate NMDA receptors; however, NT release evoked by presynaptic NMDA can be observed even in basal conditions (without depolarization), especially if physiological solutions that are free of Mg^2+^ ions are used (see [91]). Although our experiments were always performed in basal (non-depolarizing) conditions with the presence of physiological concentrations of Mg^2+^ ions, we cannot completely exclude the involvement of presynaptic NMDA. On the other hand, the effect of D-Asp was almost completely prevented by DL-TBOA (Figure 5, bottom), a blocker of EAATs that is reported as being free of activity at Glu receptors [92]. Since the effect of D-Asp is totally prevented by such a compound that exhibits selectivity for Glu transporters, we suggest that the initial targets of D-Asp are represented by EAATs, while NMDA receptors should not be involved, at least at the initial stage. Further details of the steps through which the whole process takes place are not within the aims of the present study, although they should merit further investigation.

### 4.5. Selective Labeling through GlyT1 or GlyT2 Does Not Permit Us to Establish GlyT1/GlyT2 Coexistence or Segregation on EAAT-Bearing Nerve Terminals

As shown in Figure 6, D-Asp can, although with slightly different efficacies, induce [^3^H]Gly release in three different experimental settings: (i) previous [^3^H]Gly uptake through GlyT1 and GlyT2 transporters (i.e., preincubation with [^3^H]Gly in the absence of GlyT1 or GlyT2 inhibitors) and subsequent exposure to D-Asp (Figure 5, top panel); (ii) [^3^H]Gly uptake exclusively through GlyT2 (i.e., preincubation with [^3^H]Gly in the presence of the selective GlyT1 inhibitor NFPS) and subsequent exposure to D-Asp (Figure 6, top panel); and, finally, (iii) [^3^H]Gly uptake exclusively through GlyT1 (i.e., preincubation with [^3^H]Gly in the presence of the selective GlyT2 blocker Org25543) and subsequent exposure to D-Asp (Figure 6, bottom). In the three conditions, the effect of D-Asp appears similar.

This is compatible, theoretically, with both the following possibilities: (i) the existence, at least in a limited number, of EAAT-expressing nerve terminals endowed with both GlyT1 + GlyT2; and (ii) the existence of a subset of nerve terminals endowed with EAATs and GlyT1 separately from terminals that are endowed with EAATs and GlyT2. Therefore, it is not possible here to understand whether functional “GlyT1 + GlyT2” both colocalize together on the same EAAT-bearing nerve terminal, or whether the two functional GlyTs are segregated. Further investigation would be required to adequately address this point.

### 4.6. Considerations on Possible Modes of Interaction between Gly and Glu

It is well known that amino acid NTs in nerve terminals are stored both in synaptic vesicles and in cytoplasmic pools; while depolarization-evoked Ca^2+^-dependent exocytosis is the best-known physiological mechanism of NT release, the cytoplasmic portion of NTs can be released by non-exocytotic mechanisms. Data from previous research indicate that the mechanisms of Glu or Gly release that are induced by “heterotransporter” activation did not occur by vesicular exocytosis while other, non-exocytotic mechanisms were involved [18,19,22,39]. These mechanisms included, although not exclusively, the known “carrier-mediated release” or “transporter reversal” [93].

When GlyTs and EAATs (which are Na^+^-dependent) are activated by extracellularly added substrates (present work), the Na^+^ ions cotransported with Gly (through the Na^+^-dependent GlyTs) or with D-Asp/Glu (through the Na^+^-dependent EAATs) are expected to contribute to conditions that facilitate, respectively, the reversal of EAATs (following activation of GlyTs) or the reversal of GlyTs (following activation of EAATs). Transporter reversal can contribute, at least in part, to the release of NTs of cytoplasmic origin.

### 4.7. Considerations on Gly–Glu Possible Cotransmission

As introduced in Section 1.2, increasing evidence supports the idea of Gly–Glu cotransmission. “Co-transmission” refers to synaptic release, from a given nerve terminal, of multiple mediators that behave as NTs [94]; this implies, among other conditions, the exocytotic release of two (or more) NTs onto postsynaptic receptors, as well as the presence of specific reuptake systems [34]; exocytotic corelease also requires previous costorage of the different NTs in the nerve terminal into vesicular pools, through vesicular transporters [94]. For example, Glu/GABA costorage and cotransmission has been investigated in detail, also in hippocampal nerve terminals [95,96,97,98]: GABA is packaged into synaptic vesicles through the vesicular VIAAT-VGAT transporter, while Glu is packaged into synaptic vesicles through VGluT transporters; interestingly, some reports seem to highlight that the number of terminals that are concomitantly positive for VGLUTs and VIAAT-VGAT is relatively low [99,100]. Even though these reports deal with Glu/GABA cotransmission, the reported colocalization of VIAAT-VGAT with VGluT vesicular transporters in the same nerve terminals in, likely small, subsets of neurons, also in the hippocampus, might be compatible with the costorage, in a limited set of terminals, of Gly and Glu in vesicular pools in this area, since the same vesicular transporter VIAAT-VGAT that transports GABA can also transport Gly. This seems to be in accordance with evidence provided by other authors (see [5]).

With regard to the present study, it is clearly not possible here to demonstrate Gly–Glu cotransmission. However, (see Section 1.2) the coexistence on the same nerve terminal of two functional neurotransmitter transporters is suggested as a marker of possible cotransmission [23]. The existence of transporters for Gly (of the GlyT1 type) and Glu on the same nerve terminal has been shown through different techniques or can be inferred, since GlyT1 is present on glutamatergic neurons [6,7,9,28,29,68,69]. The present study highlights and confirms the coexistence of functional GlyTs and EAATs in a subset of hippocampal nerve terminals, and we propose that it adds some further functional evidence to the possible Gly–Glu cotransmission.

- Gly, either released by the “glutamatergic-glycinergic nerve terminals” proposed here or originating from other sources (neighboring neurons, glial cells, metabolic sources…), is a substrate of the Na^+^ -dependent GlyT1 and GlyT2 transporters, which can deliver Gly to the intraterminal space; thus, the amino acid can be stored in the same terminal with Glu. The Na^+^/Gly cotransport triggers internal events leading to an increased release of Glutamate (gray and gray-dotted arrows) through mechanisms including the facilitation of EAAT reversal and other, likely, non-exocytotic mechanisms (thick black arrows; see Section 4.6).
- D-Aspartate or endogenous Glutamate activates EAATs (including transporters of the EAAT_2_ type) that are Na^+^-dependent. The cotransport of the excitatory amino acid and Na^+^ triggers presynaptic events (white arrow and black dotted arrow), leading to an increase in Gly release that can occur through mechanisms including, possibly, the reversal of GlyTs (“crossed out” arrows).
- The most speculative concepts illustrated in the figure are pointed out in gray, alongside a “question mark”. The possible Gly–Glu cotransmission would be compatible with the costorage of Gly and Glu in synaptic vesicles through VIAAT-VGAT and vGluT vesicular transporters, respectively (see Section 4.7). The cotransmitters could then be coreleased as coagonists onto postsynaptic NMDA receptors. A final demonstration of these concepts (presented in the “C” paragraph) will require further investigation.

Parts of Figure 7 were drawn by using and modifying pictures from Servier Medical Art. Servier Medical Art by Servier is licensed under a Creative Commons Attribution 3.0 Unported License (https://creativecommons.org/licenses/by/3.0/ accessed on 13 November 2023).

## 5. Conclusions

The conclusions from the present work (see the end of Section 1.3) permit us to propose a scheme (still partially speculative) showing how Gly and Glu could interact at the presynaptic level in the hippocampus, being possibly present together as cotransmitters at least in a limited number of nerve terminals (Figure 7 and legend). Independently of cotransmission, some Gly- and Glu-releasing terminals can be close to each other, and functional interactions between the two NTs can occur through their transporters contributing to the regulation of release with a reciprocal tuning of Gly- and Glu-mediated neurotransmission. This may have physiological relevance in the coagonist action of the two NTs at NMDA receptors.

It must be considered that two different NMDA coagonists exist, namely, Gly and D-serine. Gly has been identified as the main coagonist of GluN2B subunit-containing NMDA receptors, which are mainly located non-synaptically or extrasynaptically [101,102]. With regard to the “synaptic” GluN2A subunit-containing NMDA receptor, D-serine rather than Gly is considered the most relevant coagonist [101], even though a relevant role of Gly in the activation of synaptic NMDA has also been clearly disclosed (see [103]).

The dysregulations of neurotransmission mediated by Gly, both as an inhibitory NT and as an NMDA coagonist, are involved in several CNS pathologies. Pharmacological manipulations, including the selective inhibition of GlyT1 and/or GlyT2 transporters, are under study to explore novel treatments of pathological conditions like schizophrenia, pain, alcohol/drug abuse, epilepsy, depression, anxiety, and stroke [4,8,9,33,104,105,106,107,108,109,110,111,112,113,114]. Considering the complex crosstalk between Gly and Glu, it can be expected that their interactions are involved in yet unclear aspects of the pharmacology of these “glycinergic” drugs, which are still under study. Besides cotransmission, which we believe is supported by our study, it is hoped that an increased understanding of Gly–Glu interactions can contribute to better knowledge of drugs acting on glycinergic targets. In this regard, it may be important to further investigate the mechanisms proposed here in other CNS regions, and similar studies could be extended to animal models of CNS pathologies in which Gly and Glu are known, or suspected, to play a role.

## Data Availability

The data presented in this study are all available in the article. Data are also available on request from the Corresponding Author.
